# Recruiting Persons With Dementia in a Memory Clinic Workflow: Lessons From a Physical Activity Trial

**DOI:** 10.1093/geroni/igaf122.1488

**Published:** 2025-12-31

**Authors:** Lijuan Yin, Woojin Song, Maria Caceres, Kyle Jennette, Elise Hu, Neil Pliskin, Jason Soble, Naoko Muramatsu

**Affiliations:** University of Illinois Chicago, Chicago, Illinois, United States; University of Illinois Chicago, Chicago, Illinois, United States; University of Illinois Chicago, Chicago, Illinois, United States; University of Illinois Chicago, Chicago, Illinois, United States; University of Illinois Chicago, Chicago, Illinois, United States; University of Illinois Chicago, Chicago, Illinois, United States; University of Illinois Chicago, Chicago, Illinois, United States; University of Illinois Chicago, Chicago, Illinois, United States

## Abstract

Recruiting diverse populations remains a significant challenge in Alzheimer’s Disease and related dementias (ADRD) research. Memory clinics (MeC) are uniquely positioned to recruit patients directly into research and promote behavioral change; however, integrating recruitment into clinical workflows is rare, with limited knowledge about feasibility. To address this gap, this study examined facilitators and challenges in recruiting patients diagnosed with mild cognitive impairment or mild dementia for a physical activity trial at a MeC serving racially and economically diverse populations. A total of 161 potential participants were identified through neuropsychological evaluations during their MeC visit or from patient records (March 2023–December 2024). Of these, 28 were enrolled (17.4% enrollment rate; ages 50-90; 61% women; 54% Black, 18% Latino). Unexpected challenges emerged, including 1) declining referrals within the same health system, linked to the recent retirement of referring physicians and the Geriatrics Department relocation; 2) moderately high no-show rates for scheduled patients; 3) difficulty reaching patients by phone. Nevertheless, several key factors facilitated successful recruitment. Neuropsychologists were able to initiate conversations with patients about study participation at the time of evaluation, leveraging their established, trusted relationships and cognitive health expertise. Motivated by the mission of improving care and health for patients with ADRD, the multidisciplinary clinic leadership provided full support; all members committed to successful study implementation. Recruitment strategies were co-developed by the clinic and research teams, adapting to emerging challenges. This study demonstrates the potential of integrating recruitment into MeC workflows and offers strategies for engaging hard-to-reach populations in ADRD research.

